# Molecular and Histologic Adaptation of Horned Gall Induced by the Aphid *Schlechtendalia chinensis* (Pemphigidae)

**DOI:** 10.3390/ijms22105166

**Published:** 2021-05-13

**Authors:** Qin Lu, Xiaoming Chen, Zixiang Yang, Nawaz Haider Bashir, Juan Liu, Yongzhong Cui, Shuxiao Shao, Ming-Shun Chen, Hang Chen

**Affiliations:** 1 Research Institute of Resource Insects, Chinese Academy of Forestry, Kunming 650224, China; luqin_19900319@163.com (Q.L.); cafcxm@139.com (X.C.); yzx1019@163.com (Z.Y.); haiderynau170@yahoo.com (N.H.B.); liujuan301@126.com (J.L.); cafkmcyz@126.com (Y.C.); shuxiashao@126.com (S.S.); 2 The Key Laboratory of Cultivating and Utilization of Resources Insects of State Forestry Administration, Kunming 650224, China; 3 Department of Entomology, Kansas State University, 123 Waters Hall, Manhattan, KS 66506, USA; mchen@ksu.edu

**Keywords:** plant-insect interaction, horned gall, transcriptome, nutrition supply, co-evolution

## Abstract

Chinese galls are the result of hyperplasia in host plants induced by aphids. The metabolism and gene expression of these galls are modified to accommodate the aphids. Here, we highlight the molecular and histologic features of horned galls according to transcriptome and anatomical structures. In primary pathways, genes were found to be unevenly shifted and selectively expressed in the galls and leaves near the galls (LNG). Pathways for amino acid synthesis and degradation were also unevenly shifted, favoring enhanced accumulation of essential amino acids in galls for aphids. Although galls enhanced the biosynthesis of glucose, which is directly available to aphids, glucose content in the gall tissues was lower due to the feeding of aphids. Pathways of gall growth were up-regulated to provide enough space for aphids. In addition, the horned gall has specialized branched schizogenous ducts and expanded xylem in the stalk, which provide a broader feeding surface for aphids and improve the efficiency of transportation and nutrient exchange. Notably, the gene expression in the LNG showed a similar pattern to that of the galls, but on a smaller scale. We suppose the aphids manipulate galls to their advantage, and galls lessen competition by functioning as a medium between the aphids and their host plants.

## 1. Introduction

Plant galls are outgrowths of various plant tissues after stimulation by organisms ranging from insects to bacteria. They are outcomes of the interaction between parasites and their host plants [1]. In contrast to normal tissues, parasites are able control the gall to suit the parasite’s needs. Thus, plant galls have long been considered an extension of the inducer’s phenotype [2]. In some species of galling bacteria, the galls’ development is based on DNA transference; however, the exact mechanism remains obscure in the case of galls induced by insects [3,4].

The galls have many specialized structures and functions to enhance parasite fitness, according to three main hypotheses. First, completely closed galls can resist the invasion of pathogenic microorganisms and the predation of natural enemies (i.e., defense hypothesis) [5,6,7]. Second, the gall is a sink of photosynthate that can accumulate and store nutrients, and then provide the necessary nutrients for the growth and development of galling insects by intercepting organic matter and the accumulation of inorganic nutrients (i.e., nutrition hypothesis) [8]. Third, the gall is a microenvironment that protects the inducers against inclement weather (i.e., microenvironment hypothesis) [9,10].

The gall offers many advantages to the inducer, and some galls also benefit the host plant. The gall can be a deterrent that protects plant leaves from chewing by herbivores because of abundant tannins, such as galls induced by *Rectinasus buxtoni* on *Pistacia palaestina* [11]. A similar phenomenon occurs in galls present in the leaflets induced by genus *Pistacia* [12]. Thus, in some cases, the gall may be considered a reward from the inducer [13]. These studies indicate that galling insects should not be simply regraded as harmful parasites, and that more complex symbiotic relationships exist between some inducers and their host plants.

“The insect galls are in a sense new plant organs because it is the plant that produces the gall in response to a specific stimulus provided by the invading insect” [14]. The metabolism of galls is different from that of normal organs and can affect the host plants [15]. The impact of galls on host plants varies widely by species under different conditions. Some gall inducers are able to stimulate the photosynthetic rates of nearby tissues by increasing sink demand [16,17]. By comparison, a few gall inducers are known to reduce photosynthetic rates of nearby tissues of host plants [18,19]. In addition to the impact on host photosynthesis, gall inducers can alter various metabolic pathways in host plants in different ways [20]. Gall inducers can also suppress or promote host defense reactions, depending on the types of gall inducer and host plant [21].

Horned galls on *Rhus chinensis* induced by the aphid *Schlechtendalia chinensis* possess a number of interesting traits, namely, they are larger, are clustered on small leaf wings, and are an important economic product containing a large quantity of tannins [22,23]. Notably, a horned gall can host thousands of aphids at the later stage of development [24], and a high concentration of CO_2_ is accumulated within the gall because of the respiration of these aphids. This CO_2_ is useful for host plants and is transported to nearby leaves to enhance their photosynthetic efficiency. Thus, the negative effect of aphid feeding is ultimately reduced. In addition, the inner surface of the horned gall is able to absorb and reuse honeydew. Due to the combination of photosynthesis and honeydew recycling, the *S. chinensis*–*R. chinensis* system is highly efficient. During the long period of coevolution between *S. chinensis* and *R. chinensis*, the horned gall has developed a complex nutrient exchange mechanism with its host plant via sophisticated metabolism [25].

In this study, we aimed to examine the molecular and histologic basis in galls for the mutualism developed between *S. chinensis* and *R. chinensis* through adaptative coevolution. Specifically, we examined changes in various metabolic pathways in galls by comparing tissues from LNG with tissues from leaves without nearby galls (LWG) via transcriptomic analyses.

## 2. Results

### 2.1. Morphological and Histologic Structures of Horned Galls

**Figure 1:**
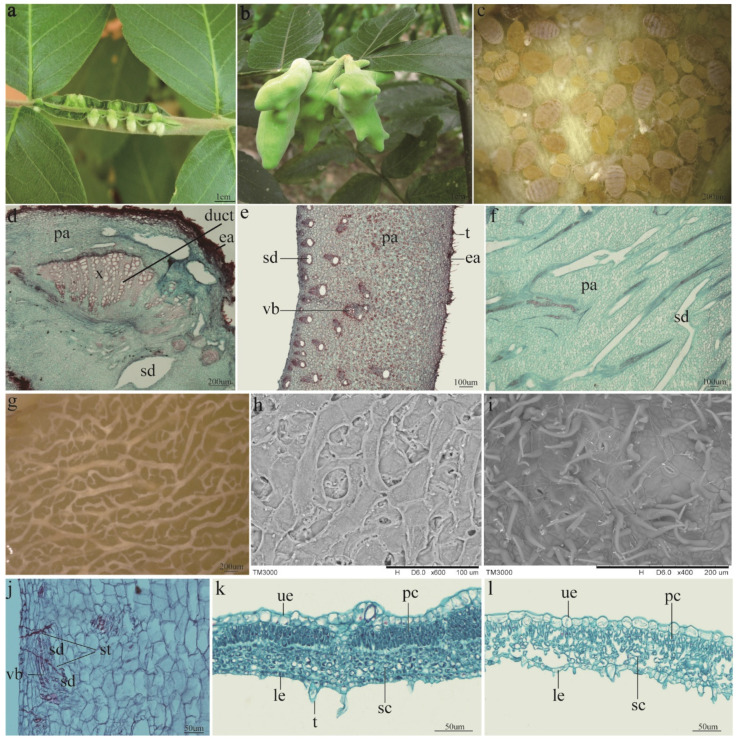
Localization, morphology, and anatomical structure of the horned gall. (**a**) Young horned galls clustered on rachis wings. (**b**) Mature galls connected with rachis leaf wing via a specialized stalk. (**c**) Aphids feeding on inner wall. (**d**) Cross-section of the stalk. Many ducts are present in the expanded xylem. (**e**) Crosscutting of the gall wall. The wall comprises parenchyma cells and contains many vascular bundles that are joined to the schizogenous ducts. (**f**) Plane surface of the horned gall; long schizogenous ducts are present. (**g**) The inner surface of the horned gall treated by NaOH. Vast, branched schizogenous ducts are present in the inner wall. (**h**) A scanning electron microscope (SEM) image of the inner surface. The inner wall was rough and characterized by holes. (**i**) The outer surface of the horned gall in the SEM image. Stoma and tomentum are present in the outer wall. (**j**) The stylophores of the aphids. Stylophores gathered in the vascular bundle of the horned gall. (**k**) The cross-section of the young *R. chinensis* leaf. The palisade and spongy cells are closely arranged. (**l**) The cross-section of the mature *R. chinensis* leaf. The cells, particularly the spongy tissues, are loose and porous. ea = epidermis-air, x = xylem, pa = parenchyma, sd = schizogenous duct, vb = vascular bundle, t = tomentum, st = stylophore, el = epidermis-lumen, pc = palisade cell, sc = spongy cell, le = lower epithelial cell, ue = upper epithelial cell.

Horned galls were located on rachis wings of the plant *R. chinensis* and induced by the aphid *S. chinensis* (Figure 1a,b). They were initially small and round, but multiple galls with irregular shapes reached a large size and could completely cover a rachis wing at a later stage (Figure 1a,b). A horned gall was connected to a rachis wing via a highly lignified base region (stalk) (Figure 1b), which had expanded xylem and ducts (Figure 1d). Aphids lived in a closed gall and fed on the inner wall of the gall (Figure 1c). Compared with galls induced by other aphids, horned galls were significantly larger and more clustered on rachis wings. A small rachis wing could carry up to 19 (12 ± 6.34) galls. To accommodate such a large number of feeding aphids, branch schizogenous ducts were present on the inner surface (Figure 1f,g) and were surrounded by densely arranged parenchyma cells and associated with vascular cells (Figure 1e). The schizogenous ducts were branched and filled the inner wall (Figure 1f,g). This was clearly different from the normal leaf structure. Tissues of the leaf showed obvious differentiation among cells, such as upper and lower epithelial cells, palisade cells, and spongy cells. The palisade and spongy cells were closely arranged in the initial stage, and they became loose and porous in the mature stage (Figure 1k,l). The inner wall was rough and characterized by a large number of holes (Figure 1h), and abundant tomentum and stoma were found on the surface of the outer wall (Figure 1i). The stylophores left by feeding aphids were present on the schizogenous ducts and vascular bundles (Figure 1j).

### 2.2. Changes in the Abundance of Selected Cofactors and Nutrients

Nicotinamide adenine dinucleotide (NAD+) is a cofactor that is central to metabolism. The abundance of NAD+, and its reduced form NADH, in addition to nicotinamide adenine dinucleotide phosphates NADP+ and NADPH, was determined in galls, LNG, and LWG (Table 1). The abundance of NAD+ and NADH was at least three-fold higher in gall tissues than in the two kinds of leaf tissues. The abundance of NADP+ was not significantly different among the different samples. However, the abundance of NADPH was more than two-fold higher in galls compared with samples from leaves with or without galls. No significant difference in the abundance of the cofactors was observed between samples from LNG and LWG, except NAD+, which was lower in LWG than in LNG.

Total proteins, fat, and starch—the three major types of energy reserves and nutrients—were also determined in galls and compared with those in leaf tissues (Table 2). Total proteins and fat were at least three-fold less in galls than in leaf-tissue samples. However, starch was significantly higher in galls than in leaf tissues. In contrast, the abundance of simple sugars including fructose, glucose, and sucrose was significantly less in galls than in leaf tissues (Table 2). No significant or marginal differences in nutrients were observed between LNG and LWG.

### 2.3. Changes in Gene Expression of Major Metabolic Pathways in Galls

To analyze the changes in gene expression of major metabolic pathways, RNA sequencing (RNA-seq) was conducted with samples from galls, LNG, and LWG. A total of 112,543 unigenes covering a total of 127,567,799 nucleotides were obtained from the combined samples. The average length of the high-quality reads was 1133 bp. High-quality reads were assembled into unigenes that covered 45 megabases with 1032 nucleotide residues (nt) in average size and N50 1746 nt. Statistics of sequence reads and assembled unigenes are shown in Table A1, Table A2, Table A3 and Table A4. Unigenes were annotated against non-redundant protein sequences (Nr), NCBI nucleotide sequences (Nt), the manually annotated and curated protein sequence database (Swiss-Prot), Kyoto Encyclopedia of Genes and Genomes Ortholog database (KEGG), and Clusters of Orthologous Groups of proteins (COG) and Gene Ontology (GO) databases. The annotation results are shown in Table A5.

**Figure 2:**
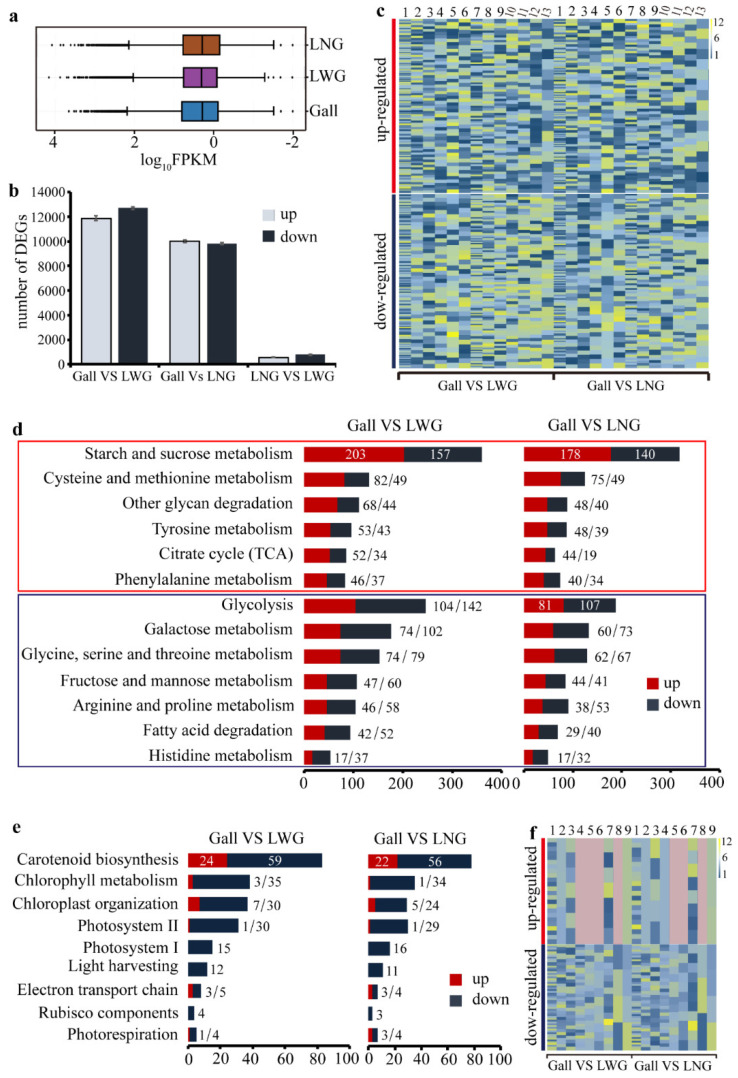
The transcriptomic analysis of the samples. (**a**) Box plot of all gene expressions. (**b**) The number of DEGs in gall vs. LWG, gall vs. LNG, and LNG vs. LWG. (**c**) Heat map of DEGs that regulate metabolism of nutrients in gall vs. LWG (log_2_ FoldChange(Gall/LWG)) and gall vs. LNG (log_2_ FoldChange(Gall/LNG)). Colors from dark blue to yellow reflect smaller to larger differences. A single block represents a single gene in the map. The numbers at the top of the graph represent different functional categories, with 1 representing starch and sucrose metabolism; 2, cysteine and methionine metabolism; 3, other glycan degradation; 4, tyrosine metabolism; 5, citrate cycle (TCA cycle); 6, phenylalanine metabolism; 7, glycolysis; 8, galactose metabolism; 9, glycine, serine and threonine metabolism; 10, fructose and mannose metabolism; 11, arginine and proline metabolism; 12, fatty acid degradation; 13, histidine metabolism. The numerical order corresponds to the ordinate from top to bottom in figure d. (**d**) DEGs involved in nutrient metabolism. The DEGs were identified in pairs of gall vs. LWG and gall vs. LNG. Up-regulated pathways are marked by red rectangles and down-regulated pathways are marked by dark blue rectangles. (**e**) DEGs involved in photosynthesis based on gall vs. LWG and gall vs. LNG comparisons. All subpathways for photosynthesis were down-regulated in gall tissues. (**f**) Heat map of DEGs related to photosynthesis based on LNG vs. LWG (log_2_ FoldChange(LNG/LWG)) comparison. The numbers at the top of the graph represent different subcategories, with 1, carotenoid biosynthesis; 2, chlorophyll metabolism; 3, chloroplast organization; 4, photosystem II; 5, photosystem I; 6, light harvesting; 7, electron transport chain; 8, rubisco components; and 9, photorespiration. Pink represents that no gene was found in this subpathway.

In terms of the number of differentially expressed genes (DEGs), LNG and LWG showed similar gene expressions, which were different from that of the gall (Figure 2a). Comparative analyses identified 11,891 unigenes were up-regulated and 12,710 unigenes were down-regulated in galls compared with LWG; 10,015 unigenes were up-regulated and 9822 unigenes were down-regulated in galls compared with LNG. In contrast, only 556 unigenes were up-regulated and 781 unigenes were down-regulated in LNG compared with LWG (Figure 2b).

Major metabolic pathways with major changes in gene expression in galls are listed in Table 3. A pathway in which two-thirds of DEGs were up- or down-regulated was considered to be significant. Seven metabolic pathways were significantly down-regulated in gall tissues compared with LWG. The down-regulated pathways included ‘photosynthesis’, ‘pentose-phosphate shunt’, ‘vitamin biosynthesis’, ‘iron-sulfur cluster and related metabolism’, ‘ATP-dependent proteases’, and ‘disease resistance proteins’. Metabolic pathways that were significantly up-regulated were also present in galls. The up-regulated pathways included ‘nucleoside biosynthesis’, ‘lipid and fatty acid transport’, ‘vesicle-mediated transport’, ‘water transport’, ‘DNA replication and repair’, ‘histones’, ‘chromosome structure and maintenance’, ‘cell cycle’, ‘cell growth’, ‘gene silencing’, ‘tissue development’, ‘structure’, ‘ribosomal proteins’, ‘translation initiation’, ‘translation elongation’, ‘response to misfolded proteins’, ‘response to metal ions’, and ‘response to salts’. The top ten genes with large differences in all pathways are listed in Table A6.

### 2.4. Gene Shift in Primary Metabolism in Galls

**Figure 3:**
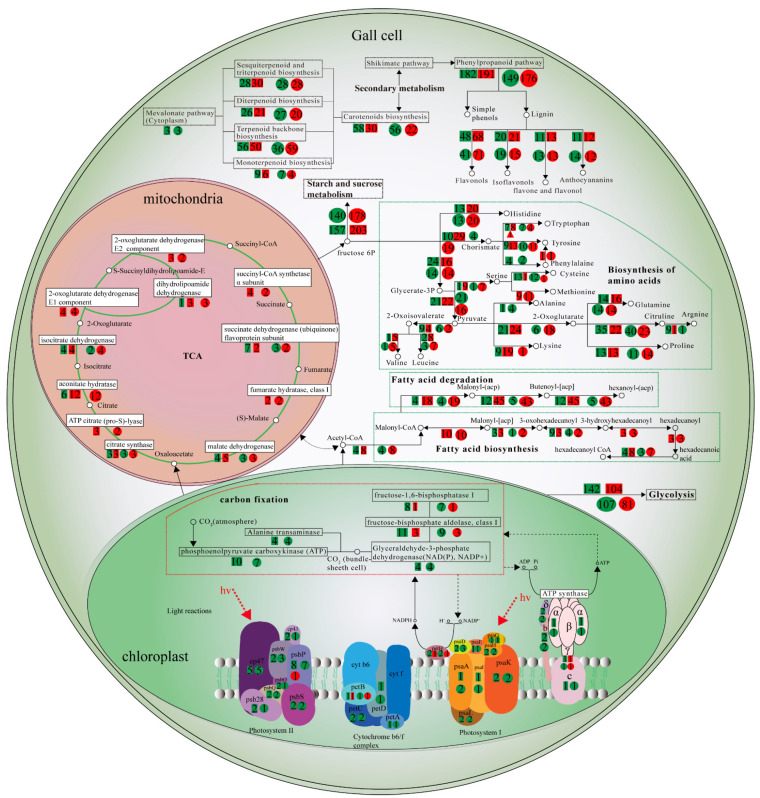
A model of the molecular adaption of horned gall cells based on transcriptomic comparative analyses. Squares indicate comparison between gall vs. LWG, circles indicate comparison between gall vs. LNG, red color indicates up-regulation, and green indicates down-regulation. Arabic numerals represent the number of genes. For photosynthesis, most subpathways were down-regulated, and phosphoenolpyruvate carboxykinase was among the most down-regulated genes for carbon fixation. Although most genes involved in the TCA cycle were up-regulated, the genes encoding succinate dehydrogenase (ubiquinone) were down-regulated. Genes for biosynthesis of essential amino acids for insects were up-regulated (histidine, leucine, lysine, methionine, tryptophan, valine), except for the genes involved in the synthesis of phenylalaine and arginine. Genes for biosynthesis of nonessential amino acids (cysteine, citruline) for insects were down-regulated. Genes involved in the production of secondary metabolites such as flavonols were generally up-regulated.

**Figure A1:**
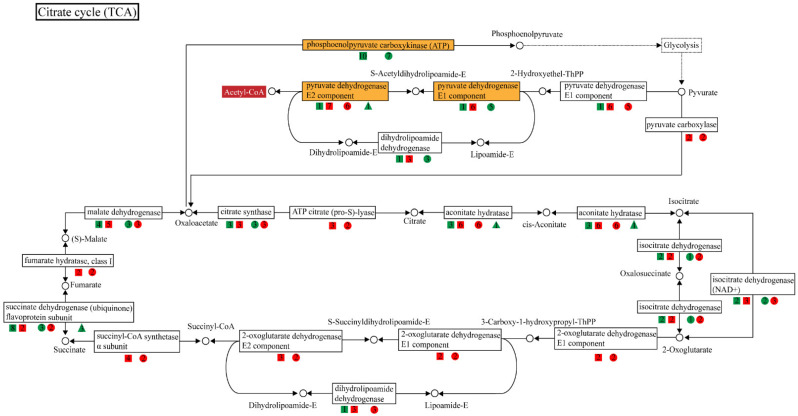
DEGs in the TCA cycle. Squares represent DEGs based on the comparison between gall vs. LWG; circles represent DEGs based on the comparison between gall vs. LNG; triangles represent DEGs based on the comparison between LNG vs. LWG. Red color indicates up-regulation and green color indicates down-regulation. The numbers within shaded shapes indicate the number of genes. There were 52 genes up-regulated and 34 genes down-regulated based on the comparison between gall vs. LWG, 44 genes up-regulated and 19 genes down-regulated based on the comparison between gall vs. LNG, and 5 genes down-regulated based on the comparison between LNG vs. LWG. The gene encoding aconitate hydratase was strongly up-regulated, but the gene encoding succinate dehydrogenase (ubiquinone) flavoprotein subunit was down-regulated in galls.

**Figure A2:**
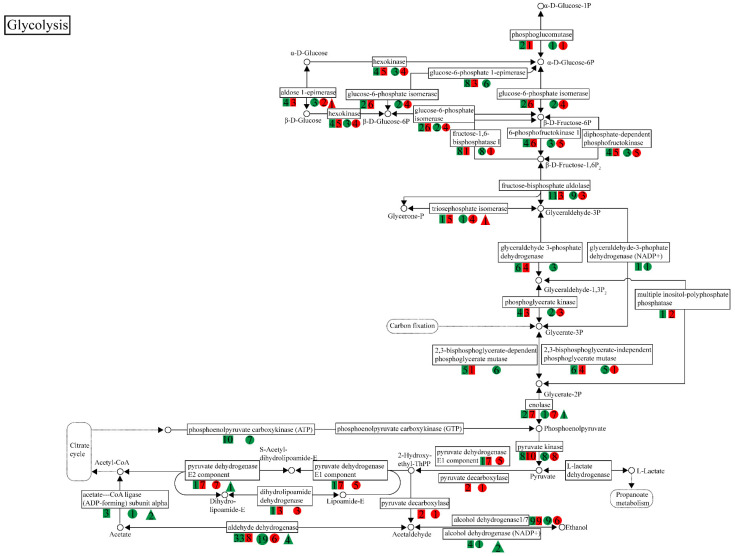
DEGs in glycolysis. Squares represent DEGs based on the comparison between gall vs. LWG; circles represent DEGs based on the comparison between gall vs. LNG; triangles represent DEGs based on the comparison between LNG vs. LWG. Red color indicates up-regulation and green color indicates down-regulation. The numbers within shaded shapes indicate the number of genes. There were 104 genes up-regulated and 142 genes down-regulated based on the comparison between gall vs. LWG, 81 genes up-regulated and 107 genes down-regulated based on the comparison between gall vs. LNG, two genes up-regulated and 11 genes down-regulated based on the comparison between LNG vs. LWG. In general, genes involved in glycolysis were down-regulated.

**Figure A3:**
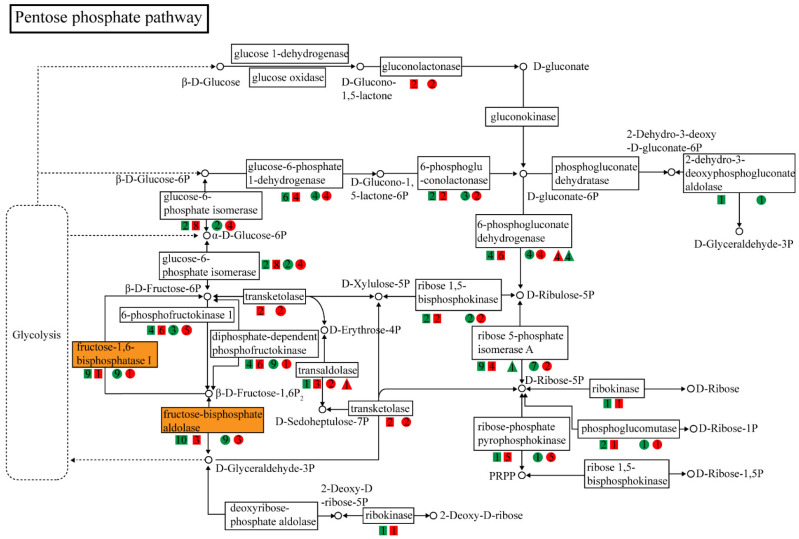
DEGs in pentose phosphate pathway. Squares represent DEGs based on the comparison between gall vs. LWG; circles represent DEGs based on the comparison between gall vs. LNG; triangles represent DEGs based on the comparison between LNG vs. LWG. Red color indicates up-regulation and green color indicates down-regulation. The numbers within shaded shapes indicate the number of genes. There were 48 genes up-regulated and 53 genes down-regulated based on the comparison between gall vs. LWG, 40 genes up-regulated and 44 genes down-regulated based on the comparison between gall vs. LNG, three genes up-regulated and three genes down-regulated based on the comparison between LNG vs. LWG. The genes encoding fructose-bisphosphate aldolase and fructose-1,6-bisphosphate I were down-regulated.

**Figure A4:**
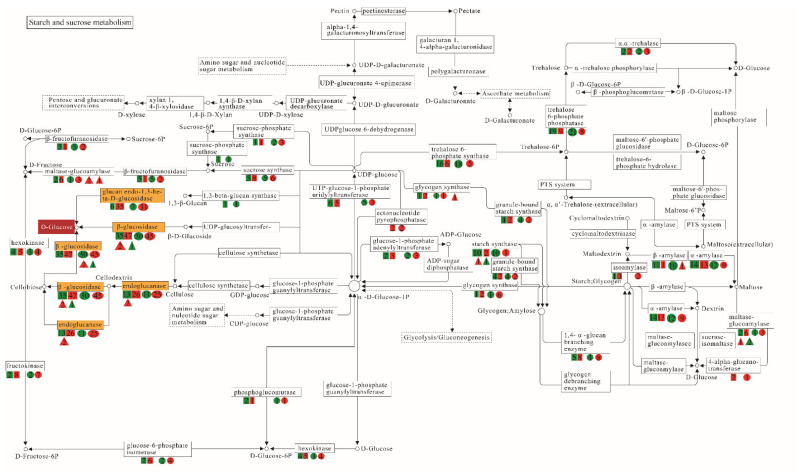
DEGs in starch and sucrose metabolism. Squares indicate DEGs based on the comparison between gall vs. LWG; circles indicate DEGs based on the comparison between gall vs. LNG; triangles indicate DEGs based on the comparison between LNG vs. LWG. Red color represents up-regulation and green color represents down-regulation. The numbers within shaded shapes indicate the number of genes. There were 203 genes up-regulated and 157 genes down-regulated based on the comparison between gall vs. LWG; 178 genes up-regulated and 140 genes down-regulated based on the comparison between gall vs. LNG; and 10 genes up-regulated and four genes down-regulated based on the comparison between LNG vs. LWG. DEGs encode β-glucosidase (47 genes up-regulated and 35 genes down-regulated in LWG vs. gall) and the glucan endo-1,3-β-d-glucosidase, both of which participate in d-glucose synthesis.

In general, more genes in the primary pathways were up-regulated than down-regulated in gall tissues (Figure 2d). The genes encoding enzymes at different steps in the tricarboxylic acid cycle (TCA cycle) were unevenly altered in galls compared with what was observed in control leaf tissues (Figure 3). For example, the aconitate hydratase encoding gene was up-regulated strongly, but the gene encoding the succinate dehydrogenase (ubiquinone) was down-regulated in galls (Figure 3 and Figure A1). The uneven alterations of gene expression in the TCA cycle result in the accumulation of simple sugars such as fructose, mannose, glucose, and galactose (Figure 2d). Consistent with increased accumulation of simple sugars, genes in glycolysis were also unevenly altered in galls. The gene encoding the aldehyde dehydrogenase was strongly down-regulated compared with the expression level in control leaf tissues (Figure A2). Similarly, the genes encoding fructose-bisphospate aldolase and fructose-1,6-bisphospatase in the pentose phosphate pathway were also down-regulated in galls, resulting in lower levels of fructose metabolism (Figure A3). The suppressed level of genes encoding the aldehyde dehydrogenase, fructose-bisphospate aldolase, and fructose-1,6-bisphospatase 1 suggested a lower level of sugar degradation. We also found that genes which regulated the ‘starch and sucrose metabolism’ and ‘other glycan degradation’ were more highly expressed in the gall tissues, and β-glucosidase regulated the last step in the starch and sucrose metabolism pathway by hydrolyzing the cellobiose or β-d-glucoside into d-glucose. In the same case, the glucan endo-1,3-β-d-glucosidase, which controlled the conversion of 1,3-β-glucan into d-glucose, also significantly increased in the gall tissues (Figure A4), which decompose the glycan into monosaccharide or disaccharide, thus benefitting aphids. The range of expressed difference is shown using a heatmap (Figure 2c).

**Figure A5:**
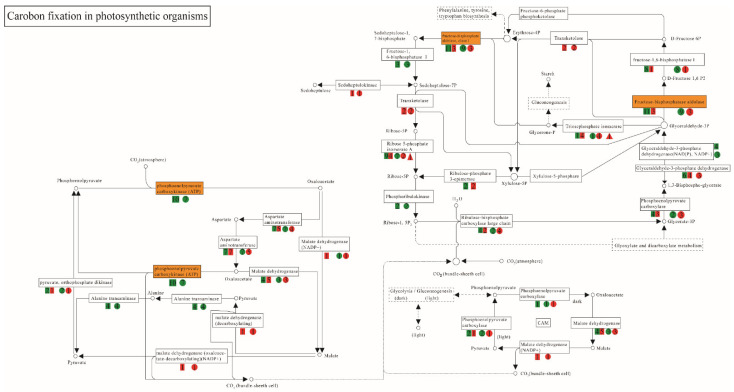
DEGs in carbon fixation. Squares indicate gall vs. LWG.; circles indicate gall vs. LNG; triangles indicate LNG vs. LWG. Red color represents up-regulation and green color represents down-regulation. The numbers within shaded shapes indicate the number of genes. There were 51 genes up-regulated and 100 genes down-regulated based on the comparison between gall vs. LWG, 46 genes up-regulated and 81 genes down-regulated based on the comparison between gall vs. LNG, two genes up-regulated based on the comparison between LNG vs. LWG. The genes encoding the first enzyme (phosphoenolpyruvate carboxykinase) in the reaction of transporting CO_2_ into cells and the enzyme fructose-bisphosphate aldolase were strongly down-regulated.

**Figure A6:**
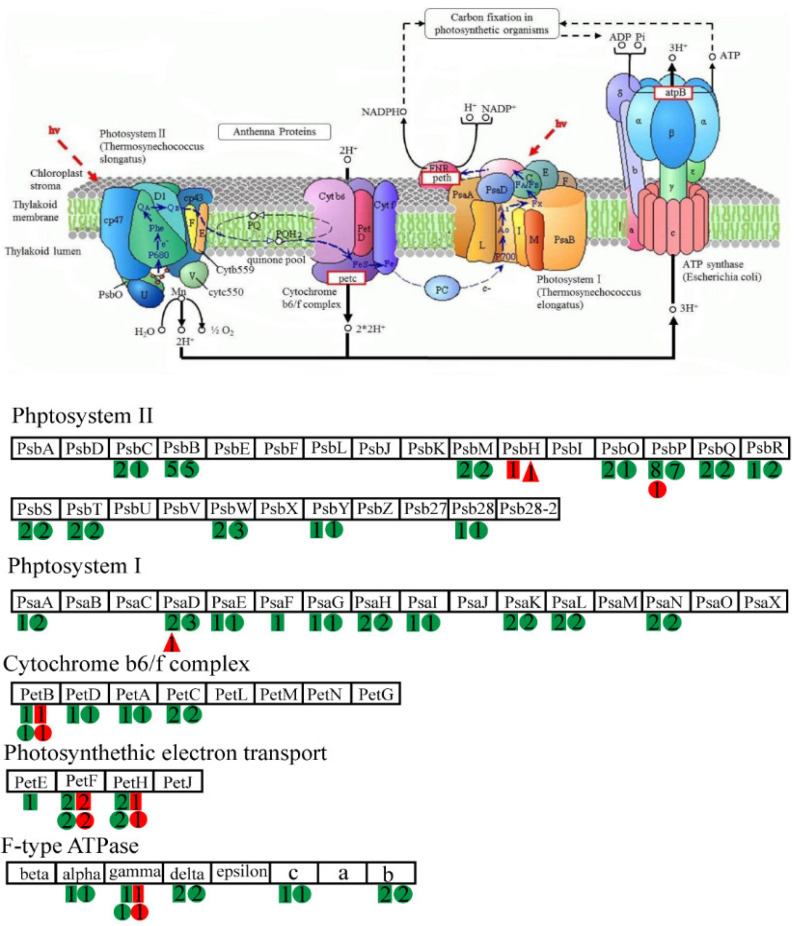
DEGs in photosystem. Squares indicate gall vs. LWG; circles indicate gall vs. LNG; triangles indicate LNG vs. LWG. Red color represents up-regulation and green color represents down-regulation. The numbers within shaded shapes indicate the number of genes. There were six genes up-regulated and 62 genes down-regulated based on the comparison between gall vs. LWG, six genes up-regulated and 61 genes down-regulated based on the comparison between gall vs. LNG, two genes up-regulated based on the comparison between LNG vs. LWG. Most genes involved in photosynthesis were down-regulated in galls.

Genes for photosynthesis were generally inhibited compared with control leaf tissues (Figure 2e and Figure A5). In particular, genes encoding components in the carbon fixation were expressed at significantly lower levels (Figure 2f, Figure 3 and Figure A6).

There was a significant shift in the biosynthesis and degradation of amino acids. Genes involved in the biosynthesis pathways of essential amino acids, such as histidine, leucine, lysine, methionine, tryptophan, and valine, were generally up-regulated in galls. In contrast, genes involved in the biosynthesis of nonessential amino acids, such as alanine and cysteine, were generally down-regulated in galls (Figure 3, Table A7). For amino acid degradation, genes involved in the degradation of cysteine, methionine, phenylalanine, and tyrosine were down-regulated in galls, whereas genes involved in the degradation of glycine, serine, threonine, arginine, and proline were up-regulated in galls (Figure 2d, Table A8).

### 2.5. Gene Shift in Secondary Metabolism in Galls

The largest differences among secondary metabolic pathways between galls and control leaf tissues were found in the genes involved in phenylpropanoid pathways (Figure 3), which are involved in plant defense. There were 18 genes involved in the metabolism of various phenylproponoids, including flavonol, isoflavonols, and anthrocyanains, which were up-regulated in galls, whereas only 9 genes were down-regulated (Table A9). In addition to the shift in gene expression involved in phenylproponoid metabolism, genes involved in terpenoid backbone and carotenoid biosynthesis were also affected (Figure 3).

### 2.6. Impact of Galls on the Metabolic Pathways in LNG

**Figure 4:**
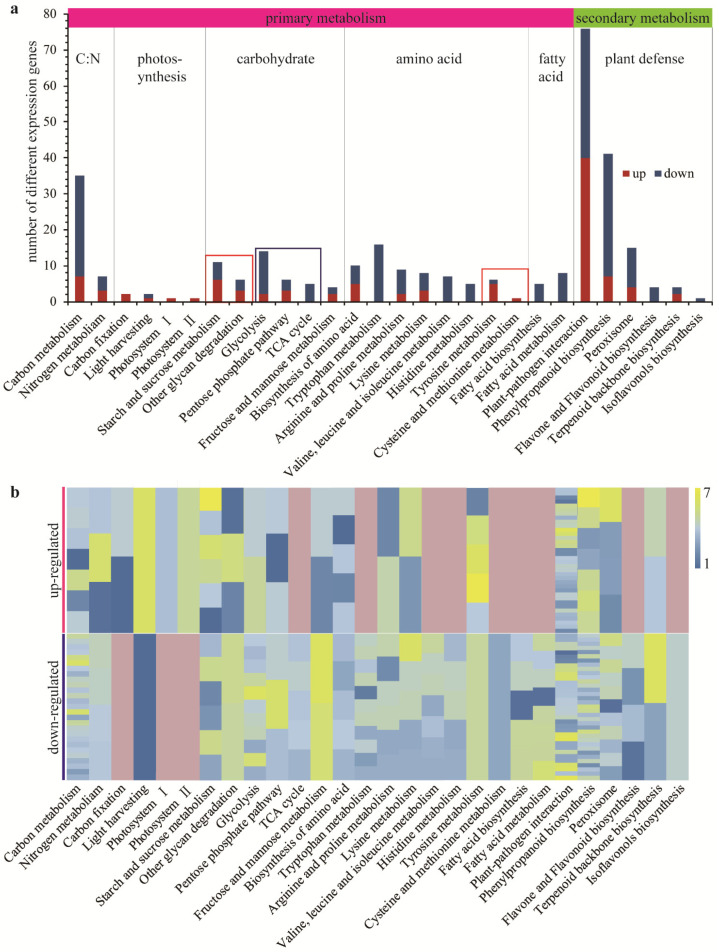
Pathways with DEGs between LNG vs. LWG. (**a**) DEGs in pathways for primary and secondary metabolism related to nutrient metabolism and plant defense. Genes involved in photosynthesis were up-regulated. Genes involved in metabolism of carbohydrates, most amino acids, fatty acids, and secondary metabolites (especially plant defense pathways) were down-regulated, except the plant–pathogen interaction. (**b**) Heatmap of DEGs. One box represents one gene. Pink color indicates no gene discovered. DEGs in LNG vs. LWG exhibited smaller differences in general compared with the differences between gall and leaf tissues.

The trend of differences in gene expression between LNG and LWG was similar to that of differences in gene expression between galls and LWG, but at a significantly smaller scale. Only 556 unigenes showed higher transcript abundance in LNG compared with LWG. Similarly, only 781 unigenes showed lower transcript abundance (Figure 2b). Genes involved in glycan metabolism were up-regulated, but monosaccharides and disaccharides were retained (Figure 4a). Genes involved in the metabolism of selected amino acids, including tyrosine, cysteine, and methionine, were up-regulated, whereas genes involved in the metabolism of arginine, proline, valine, tryptophan, leucine, and isoleucine were down-regulated (Figure 4a). Genes involved in fatty acid metabolic pathways and secondary metabolism were also down-regulated. Photosynthesis was slightly up-regulated (Figure 4a). The highest number of DEGs was present in plant–pathogen interaction pathways, but the number of down-regulated unigenes was close to that of up-regulated unigenes; the major changes in DEGs are listed in Table A10.

### 2.7. Selective Expression Genes in the Gall and LNG

**Figure 5:**
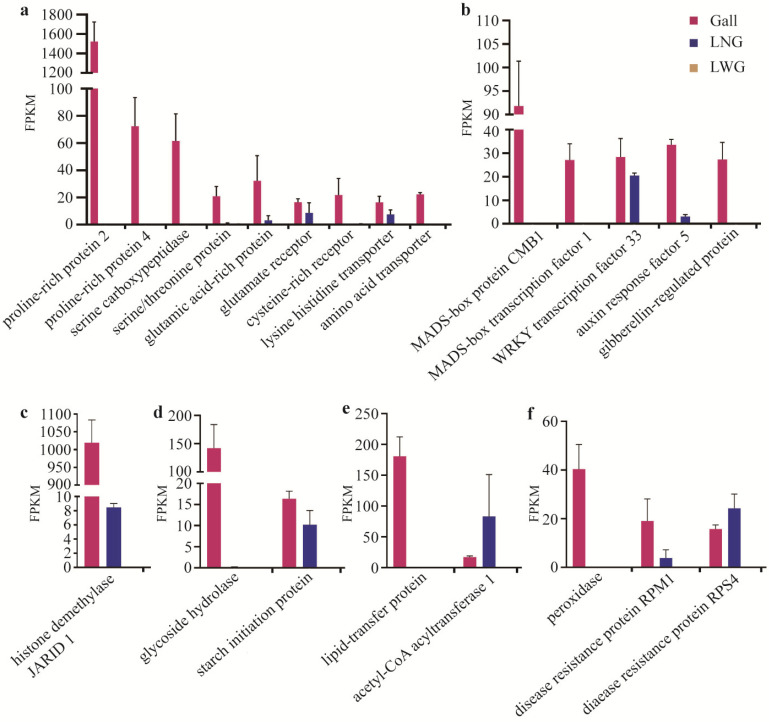
Selective expression genes in the gall and LNG. (**a**) The selective expression unigenes regulated the amino acid pathway. The unigene encoding proline-rich protein 2 showed the highest expression level in the gall. (**b**) The selective expression unigenes regulated the plant development. (**c**) The selective expression unigenes regulated the DNA methylation. (**d**) The selective expression unigenes regulated the sugar pathway. (**e**) The selective expression unigenes regulated the lipid pathway. (**f**) The selective expression unigenes regulated the plant resistance. Most unigenes expressed in the gall only, some unigenes expressed in the gall and LNG, and all unigenes could not be detected in the LWG.

A more important finding was that unigenes involved in amino acid, plant development, DNA methylation, sugar pathway, lipid pathway, and plant resistance showed selective expression in LNG and the gall. (Figure 5). In the amino acid pathway, the unigene that encodes proline-rich protein 2 showed a significant difference; its FPKM in the gall was 1523.48, but it could not be detected in LNG and LWG. The unigenes that regulate proline-rich protein 4, serine carboxypeptidase, cysteine-rich receptor, and amino acid transporter showed the same tendency. The unigenes encoding glutamic acid-rich, glutamate receptor, lysine histidine transporter, and serine/threonine-protein could be detected in the gall and LNG, but they did not express in the LWG (Figure 5a). In the plant growth pathway, three unigenes were expressed in the gall only, which regulate the MADS-box and gibberellin-regulated protein. The unigenes encoding WRKY transcription factor 33 and auxin response factor 5 were expressed in the gall and LNG (Figure 5b). The unigenes regulating the glycoside hydrolase and starch initiation protein were also expressed in the gall and LNG, but the expression level of glycoside hydrolase was lower in the LNG (Figure 5d). In the lipid pathway, the unigene that encodes lipid-transfer protein could be detected in the gall only; the unigene regulating the acetyl-CoA acyltransferase 1 showed a different expression pattern, and the expression level in the LNG was higher than that in the gall (Figure 5e). Aphids also caused changes in plant resistance. The unigene that encoded peroxidase was only expressed in the gall, and the disease resistance proteins RPM1 and RPS4 could be detected in the gall and LNG; however, the expression level of RPS4 in LNG was higher than that in the gall (Figure 5f). Notably, we found that a unigene that regulated the histone demethylase JARID1 was only expressed in the gall and LNG, and the FPKM in the gall was significantly higher than that in LNG (Figure 5c). The sequences of selective unigenes are listed in Appendix A.

## 3. Discussion

Several characteristics of horned galls on *R. chinensis* induced by *S. chinensis* are unique and notable. For example, a significant number (12 ± 6.34) of galls with large size (86.69 ± 13.15 × 53.63 ± 12.74 mm) is exclusively localized on tiny rachis wings instead of being present on bigger leaves. A gall can carry tens of thousands of aphids, and green galls are strong candidates to perform photosynthesis [24,25]. To gain insight into these unique characteristics, we conducted comparative analyses of gene expression on the differences and similarities of metabolic pathways between galls and leaf tissues. We found several unique shifts in metabolic pathways in galls in comparison with normal leaf tissues. One of the shifts was the unbalanced changes in gene expression involved in the TCA cycle. For example, the gene encoding aconitate hydratase was up-regulated, yet the gene encoding succinate dehydrogenase was down-regulated. TCA is the central metabolic pathway of carbohydrates, lipids, and proteins [26]. The unbalanced shift of the TCA cycle might be responsible for reducing the metabolism of simple sugar and enhanced accumulation of NAD+/NADH. Aphids can easily utilize simple sugar and NAD+/NADH as nutrients. Similarly, glycolysis and pentose phosphate pathways also exhibited an unbalanced shift, favoring accumulation of simple sugar and degradation of polysaccharides and other macromolecules such as lipids and proteins. Low concentrations of glucose, fructose, sucrose, and fat were found in horned gall tissues, although biosynthesis of monosaccharides was enhanced. This is to be expected because aphids are likely to use these substances, whereas it is difficult for them to use starch directly because of their digestive system [27]. Starch is a nutrient that breaks down into glucose during the period of nutritional deficiency [28]. It is interesting to note that similar changes in gene expression were also observed in LNG compared with LWG, although the changes were at much smaller scales.

Aphids are phloem feeders; however, only a smally number of amino acids in plant phloem can be obtained, meaning that aphids’ demand for amino acids cannot be met [29]. Our transcriptomic analyses revealed that the galls provide solutions for this problem: the genes involved in the synthesis of essential amino acids for aphids were up-regulated in galls compared with those in normal leaves. The up- and down-regulation of selective genes are likely due to fulfilling the nutrient requirement of aphids during the long period of host–aphid coevolution. The contents of free and essential amino acids in the gall and LNG are significantly higher than those in LWG (Table A11) [30]. The molecular mechanism of this selected gene regulation in galls remains to be determined. Moreover, 99% of *S. chinensis* symbiotic bacteria are *Buchnera* spp., the main contributors of amino acids for aphids [31]. We suppose the enhanced biosynthesis pathways of essential amino acids compensate for the lack of amino acids synthesized by *Buchnera* spp.

Changes in expression levels were also found in genes involved in secondary metabolism in galls. The most interesting change in secondary metabolism was the up-regulation of most of the genes involved in phenylproponoid metabolism. Phenylproponoids are toxic to insects and microbes. Aphids may use phenylproponoids as a defense chemical for potential secondary infection from microbes, whereas the aphids themselves have adapted to these toxic chemicals during the long period of coevolution. The up-regulation of phenylproponoid genes is in contrast to most other defense genes, such as jasmonic acid and ethylene pathways, which were generally not significantly different.

A remarkable finding was that some of the selective expressed unigenes in the gall and LNG played a wide range of regulatory roles in plants, such as enhancing the enrichment and transportation of amino acids and lipids, and enhancing glycoside hydrolysis and biosynthesis of starch. These roles help to provide more suitable nutrients and standby nutrient storage. The unigenes regulating auxin and gibberellin were highly expressed in the gall, and the identified content of auxin in the gall was higher than that in normal tissues [32]. The volume of the horned gall grows rapidly with the population growth of the aphids from July to August [24]. The unigenes regulating the histone demethylase JARID1 showed the second highest FPKM (1019.54) in the gall. DNA methylation is an important regulatory form of epigenetics and can affect a large number of biological processes, such as plants’ resistance and flowering phase [33]. This indicates that the molecular regulation mechanism of the gall is more complex, and DNA methylation is an ignored factor. In general, more unigenes were selectively expressed in the gall; some of these were also expressed in LNG, but showed significantly less FPKM. We suggest that the horned gall functions as a comfortable shelter according to complex molecular regulation, and the gall affects the metabolism of nearby tissues.

Other changes in gene expression in galls include the up-regulation of genes involved in DNA synthesis, cell division, tissue development, and other structural proteins. The up-regulation of these genes may provide the basis for continuous growth of galls through the growing season (from May to October), that provides enough space for aphids. The shifts observed in primary metabolic pathways were also reported in some other gall systems [34,35]. However, a recent survey on grape leaf galls induced by *Daktulosphaira vitifoliae* suggested that genes involved in primary metabolic pathways including glycolysis and the citric acid cycle were up-regulated, resulting in a metabolic shift from autotrophic to heterotrophic [15]. Many factors can affect the functioning of plant galls, including the types of galling parasites, and the location and shape of a gall [5,36,37]. The contradictory observations among different gall systems may reflect different strategies to achieve harmonic coexistence between gall inducers and host plants.

In addition to the molecular adaption, the specialized histologic structure also plays a key role in the interaction between *R. chinensis* and *S. chinensis*. The gall wall comprises parenchyma cells, which not only store a large quantity of nutrients such as starch, but also contain a high level of tannins, which reduce the feeding times of herbivores [11]. Furthermore, the expanded xylem in the stalk can provide enough space for nutrient exchange between the aphids and their host plants. This also provides strong mechanical support via a tight connection to the host plant; a horned gall was found to weigh 10.25 ± 3.67 g and contain 19,850.11 ± 559.43 aphids in the latter stage of the gall development. [24]. Another remarkable characteristic of the horned gall is the presence of vast branched schizogenous ducts that are associated with the vascular bundles in the wall. These form a net that wraps the aphids. The distribution of the schizogenous ducts is regular. The average diameter of schizogenous ducts in the inner wall (3.31 ± 1.97 μm) was much smaller than those in the outer wall (6.05 ± 2.34 μm), but schizogenous ducts in the inner wall (9.28 ± 5.35/mm^2^) were more abundant than in the outer wall (0.15 ± 0.27/mm^2^) [38]. These branched structures increase the aphids’ contact surface with the gall and improve the efficiency of transportation and exchange of nutrients. Thus, the stylophores gather in the schizogenous ducts.

**Figure 6:**
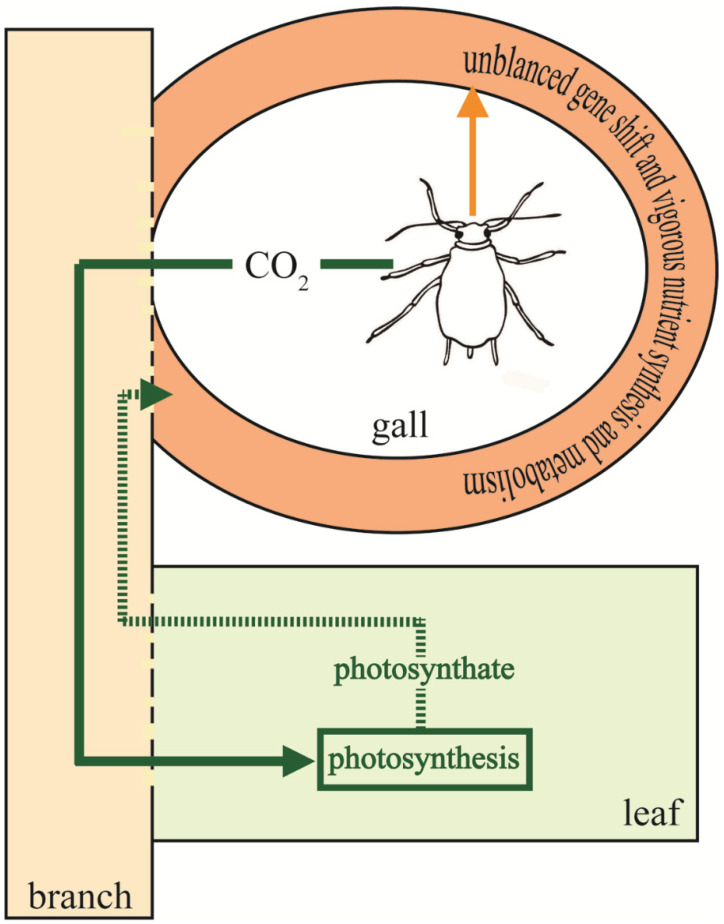
A model for mutualism between aphids and *R. chinensis*. Host plants provide aphids with photoassimilates, minerals, and other nutrients. The aphids can control the horned gall and the host plant for the aphids’ own benefit, but the horned gall eases the direct conflict by unbalanced gene shifting and vigorous nutrient biosynthesis and metabolism. Aphids generate a high concentration of carbon dioxide, which can elevate photosynthesis of nearby leaf blades. In addition, aphids may also perform nitrogen fixation and other benefits for host plants. Due to the mutualism, leaves with multiple galls usually outgrow leaves without galls despite aphid feeding.

The gall inducer can control the host plant for the inducer’s benefit; thus, the gall is considered an extended phenotype of the parasite [39]. The similarity in gene expression between galls and neighboring host tissues suggests aphids induce changes in remote host tissues. However, the gall bears the majority of the stress caused by aphids by shifting its gene expression. In addition, the levels of CO_2_ in galls are much (on average 8–16 times) higher than that of atmospheric CO_2_. High CO_2_ within the gall could be delivered to the gall tissues and nearby leaves, and thereby enhance rates of photosynthesis [25]. Thus, feeding aphids may not have a negative effect on the trees (Figure 6).

## 4. Materials and Methods

### 4.1. Materials

**Figure 7:**
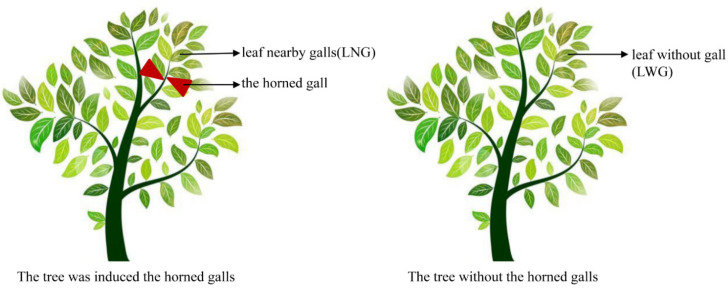
The samples in this study.

The aphids *S. chinensis* were reared on *R. chinensis* trees in the green house of the Research Institute of Resource Insects of Kunming, Yunnan province, southwest China. The samples were collected on 16 August 2019. After removing aphids from the gall tissues, the collected galls were divided into four parts. One part was frozen in liquid nitrogen and stored at −80 °C for later isolation of RNA. Another part was used for determining the content of proteins, fats, starch, fructose, glucose, sucrose, NADP+, NADPH, NAD+, and NADH. The third part was fixed immediately in an FAA solution (5 mL formaldehyde, 5 mL acetic acid, 90 mL 70% ethyl alcohol) for 5 days and 4% glutaraldehyde for two hours, respectively. The remaining parts were cut into 5 × 5 mm and placed in 50% NaOH for two days. At the same time, LNG and LWG were collected and similarly treated (Figure 7). To reduce sampling error, each sample was mixed from more than five galls or leaves from different trees. Each test had three biological replications.

### 4.2. Tissue Anatomy

The samples in a FAA solution of the galls were cut into 2–3 mm pieces and then dehydrated in an ethanol series (70% ethyl alcohol for 30 min, 80% ethyl alcohol for 20 min, 90% ethyl alcohol for 15 min, 95% ethyl alcohol for 10 min, and 100% ethyl alcohol for 5 min). Then, ethyl alcohol was replaced by xylene and paraffin in turn. The gall samples were embedded in paraffin, 16-μm-thick sections were made using a rotary microtome (Leica RM2126RT, Solms, Hesse-Darmstadt, Germany), and the leaves were cut into 4-μm-thick sections. The sections were de-paraffinized and stained with safranin and fast green after parching.

The fixed samples in 4% glutaraldehyde were dehydrated in an ethanol series (30% ethyl alcohol for 30 min, 50% ethyl alcohol for 30 min, 70% ethyl alcohol for 30 min, 80% ethyl alcohol for 20 min, 90% ethyl alcohol for 15 min, 95% ethyl alcohol for 10 min, and 100% ethyl alcohol for 5 min) and dried in air. The dried samples were sprayed gold and observed under a SEM (Tabletop Microscope 3000, Tokyo, Japan).

The horned galls were observed via a three-dimensional microscope after treatment by 50% NaOH (MSD-VHX1000, Tokyo, Japan).

### 4.3. Coenzyme Measurements

The determination of coenzyme was carried out using an NAD/NADH Quantitation Colorimetric Kit (Biovision, San Francisco, CA, USA) and an NADP/NADPH Quantitation Colorimetric Kit (Biovision, San Francisco, CA, USA). The assays were performed following the instructions of the kits.

### 4.4. Nutrients

Protein contents were determined using the Kjeldahl nitrogen determination method (FOSS kjeltecTM 2300, Hoganas, Sweden). Fat was measured using the Soxhlet extraction method (Rotavapor^®^ R-II BUCHI, Hoganas, Sweden). Starch was determined using the enzyme hydrolysis method (Varioskan Flash, Waltham, MA, USA). Fructose, glucose, and sucrose were measured using liquid chromatography (High-Performance Liquid Chromatograph Ultimate 3000, Waltham, MA, USA).

### 4.5. Illumina Sequencing and Transcriptome Analysis

Total RNA was extracted from 1 mg of tissues using an RNA Extraction Kit (BioTeke Corporation, Beijing, China) according to the manufacturer’s protocols. RNA purity and integrity were assessed using an RNA Nano 6000 Assay Kit (Agilent Technologies, Palo Alto, CA, USA). First strand cDNA was synthesized with random hexamers as primers and mRNA fragments as templates. Double-stranded cDNA was synthesized and purified using a QiaQuick PCR Extraction kit (QIAGEN, Dusseldorf, North Rhine-Westphalia, Germany). End repair and the addition of adenines were carried out in the EB buffer. Sequencing adaptors were ligated to fragments, and the resulting fragments were purified through agarose gel electrophoresis and enriched by PCR amplification. Sequencing libraries were generated using a Next Ultra Directional RNA Library Prep Kit from Illumina (New England Biolabs, Beijing, China). After cluster generation, the libraries were sequenced on an Illumina HiSeq 2000 platform (BGI, Shenzhen, Guangdong, China), and paired-end reads were generated. Finally, products were purified with the AMPure XP system (Beckman Coulter, Brea, CA, USA), and library quality was confirmed on an Agilent 2100 Bioanalyzer (Agilent Technologies, Palo Alto, CA, USA).

Reads with adaptors or more than 10% unknown bases, in addition to other low-quality reads, were removed prior to data analysis. Transcriptome assembly was accomplished using Trinity with default parameters [40]. The sequences were assessed by read quality, statistics of alignment analysis, sequencing saturation analysis, distribution of reads on the reference gene, and distribution of reads on the reference genome.

### 4.6. Gene Annotation

Gene function of all assembled unigenes was annotated based on the Nr, Nt, Swiss-Prot, KEGG, COG, and GO databases.

### 4.7. Differentially Expressed Genes (DEGs)

Based on the gene expression level (FPKM), we identified the DEGs among samples, and the DEGs of gall vs. LWG, gall vs. LNG, and LNG vs. LWG were calculated by log_2_ fold change (Gall/LWG, Gall/LNG, and LNG/LWG), respectively. Fold change of >1 indicated up-regulation, whereas a negative fold change indicated down-regulation.

## 5. Conclusions

Our study revealed extensive gene shifts in primary and secondary metabolic pathways in *R. chinensis* horned galls induced by the aphid *S. chinensis*. Most shifts in gene expression appeared to be driven by adaptation to satisfy nutrient needs of aphids. The complex metabolism of photosynthesis in gall tissues is different from that of normal tissues. Genes that accommodate the aphids were selectively expressed in the gall and LNG, and indicate DNA methylation is an ignored regulatory factor in the gall. Furthermore, the horned galls have specialized histological structures such as branched schizogenous ducts and expanded xylem. Due to its molecular foundation and histological adaption, the horned gall is a medium that eases direct conflict between the aphids and the host plant, so the normal physiological state of *R. chinensis* can be maintained. As a result, galling aphids have formed a relatively harmonic co-existence with their host plants via partially self-supporting galls.

## Figures and Tables

**Table 1:** The content of coenzymes in the different samples.

Coenzymes	Gall	LWG	LNG
NAD^+^ (nmol\g)	142.74 ± 9.17 ^a^	30.57 ± 1.28 ^c^	42.62 ± 6.68 ^b^
NADH (nmol\g)	108.75 ± 9.94 ^a^	27.07 ± 2.29 ^b^	30.35 ± 6.44 ^b^
NADP^+^ (nmol\g)	12.46 ± 2.01 ^a^	12.13 ± 0.49 ^a^	11.72 ± 0.37 ^a^
NADPH (nmol\g)	36.77 ± 11.21 ^a^	15.15 ± 1.48 ^b^	15.86 ± 0.92 ^b^

Note: different letters in the same row represent the significant difference at the level of *p* < 0.05.

**Table 2:** The percentage of different types of nutrients in dried tissues.

Nutrients	Gall	LWG	LNG
Protein (%)	3.28 ± 0.18 ^b^	11.18 ± 0.03 ^a^	11.19 ± 0.09 ^a^
Fat (%)	1.64 ± 0.13 ^b^	3.61 ± 0.18 ^a^	3.83 ± 0.37 ^a^
Starch (%)	2.51 ± 0.06 ^a^	1.83 ± 0.07 ^b^	1.02 ± 0.05 ^c^
Fructose (%)	0.91 ± 0.04 ^c^	3.31 ± 0.03 ^a^	2.52 ± 0.04 ^b^
Glucose (%)	0.94 ± 0.001 ^c^	2.17 ± 0.03 ^a^	1.82 ± 0.001 ^b^
Sucrose (%)	1.29 ± 0.02 ^b^	2.50 ± 0.12 ^a^	2.40 ± 0.02 ^a^

Note: different letters in the same row represent the significant difference at the level of *p* < 0.05.

**Table 3:** The up- and down-regulated pathways in gall vs. LWG (based on KEGG pathway).

Pathways	Functional Category/Subcategory	Total	Down-Regulated	Up-Regulated	Down/Up
Photosynthesis	Photosynthesis	233	194	39	4.97
Pentose phosphate pathway	Pentose-phosphate shunt	114	78	14	5.57
	Vitamin	29	14	4	3.50
	Iron and sulfate metabolism	47	27	3	9.00
	small molecule metabolism	100	49	18	2.72
	Disease resistance proteins	56	18	9	2.00
	ATP-dependent proteases	27	15	2	7.50
	Cysteine proteases	13	6	3	2.00
Metabolism	Nucleoside metabolism	159	22	59	0.37
	Structure	236	27	129	0.21
Protein synthesis	Ribosomal proteins	193	29	128	0.23
	Translation	275	56	140	0.40
	Initiation	85	16	44	0.37
	Elongation	33	2	17	0.12
	Others	123	22	70	0.31
	Protein myristoylation	39	6	15	0.40
Transport	Lipid and fatty acid transport	60	10	20	0.50
	vesicle-mediated and introcellular transport	105	13	50	0.26
	Water transporters	4	3	1	3.00
	Gene silencing	124	20	41	0.49
DNA replicaiton and cell cycle	DNA repair	278	37	84	0.44
	DNA replication	113	12	38	0.32
	DNA modification	19	0	6	0
	Other DNA metabolic processes	23	4	8	0.50
	Histones	113	21	47	0.45
	Chromasome structure maintenance	82	11	29	0.38
	Cell cycle regulation	292	31	113	0.27
Development	Cell growth	154	15	72	0.21
	Tissue development	413	70	167	0.42
	Other developmental processes	164	23	53	0.43
Stress response	Response to metal	81	12	39	0.31
	Response to misfolded proteins	29	6	14	0.43
	Response to salts	89	15	36	0.42

## Data Availability

The data that support the findings of this study are openly available in the NCBI Sequence Read Archive (SRA) under projects PRJNA631065 (https://www.ncbi.nlm.nih.gov/bioproject/PRJNA631065. Access worked from 7 May 2020).

## Appendix A.

**Table A1:** Statistics of transcriptome.

	Sample	Total Number	Total Length (bp)	Mean Length (bp)	N50	GC%
**Unigene**	Rhuscm	112,543	127,567,799	1133	2061	40.34

Sample: Sample name; Total Number: The total number of transcripts; Total Length: The read length of transcripts; Mean Length: The average length of transcripts; N50: a weighted median statistic that 50% of the total length is contained in Unigenes that are equal to or larger than this value; GC(%): the percentage of G and C bases in all transcripts.

**Table A2:** Quality evaluation of clean reads.

Samples	Total Raw Reads (Mb)	Total Clean Reads (Mb)	Total Clean Bases (Gb)	Q20 Percentage (%)	Q30 Percentage (%)	Clean Reads Ratio (%)
Gall-1	45.72	44.23	6.63	98.27	95.56	96.73
Gall-2	45.72	44.11	6.62	98.27	95.56	96.47
Gall-3	45.72	44.07	6.61	98.25	95.51	96.39
LWG-1	47.36	45.53	6.84	98.15	95.28	96.15
LWG-2	47.36	45.60	6.83	98.19	95.38	96.28
LWG-3	47.36	45.56	6.84	98.22	95.44	96.21
LNG-1	47.36	45.61	6.84	98.29	95.60	96.31
LNG-2	45.72	44.04	6.61	98.32	95.69	96.33
LNG-3	47.36	45.61	6.84	98.29	95.61	96.31

Sample: Sample name; Total Raw Reads(Mb): The reads amount before filtering; Total Clean Reads(Mb): The reads amount after filtering; Total Clean Bases(Gb): The total base amount after filtering; Clean Reads Q20(%): The rate of bases which quality is greater than 20 value in clean reads; Clean Reads Q30(%): The rate of bases which quality is greater than 30 value in clean reads; Clean Reads Ratio(%): The ratio of the amount of clean reads.

**Table A3:** Quality evaluation of transcripts.

Samples	Total Number	Total Length	Mean Length	N50	N70	N90	GC (%)
Gall-1	59,801	52,964,643	885	1663	996	318	41.02
Gall-2	66,917	57,745,662	862	1603	938	313	41.19
Gall-3	57,263	48,501,165	846	1552	915	308	41.72
LWG-1	82,726	60,330,352	729	1435	717	258	40.20
LWG-2	63,813	54,324,268	851	1598	942	305	40.77
LWG-3	68,304	55,058,251	806	1545	875	282	40.41
LNG-1	59,628	51,521,169	864	1602	956	311	41.05
LNG-2	59,609	53,137,603	891	1646	1000	326	40.52
LNG-3	59,127	52,809,348	893	1621	995	329	40.45

Sample: Sample name; Total Number: The total number of transcripts; Total Length: The read length of transcripts; Mean Length:The average length of transcripts; N50: The N50 length is used to determine the assembly continuity, the higher the better, N50 is a weighted median statistic that 50% of the total length is contained in Unigenes that are equal to or larger than this value; N70: Similar to N50; N90: Similar to N50; GC(%): the percentage of G and C bases in all transcripts.

**Table A4:** Quality evaluation of unigenes.

Samples	Total Number	Total Length	Mean Length	N50	N70	N90	GC(%)
Gall-1	42,432	45,357,023	1068	1810	1190	422	41.16
Gall-2	48,483	49,794,838	1027	1745	1118	403	41.33
Gall-3	41,018	41,199,383	1004	1703	1093	389	41.84
LWG-1	54,078	49,698,140	919	1656	1002	332	40.52
LWG-2	43,523	45,592,506	1047	1760	1152	421	40.89
LWG-3	46,052	46,151,536	1002	1727	1117	383	40.64
LNG-1	41,269	43,554,246	1055	1758	1162	428	40.14
LNG-2	41,022	44,757,790	1091	1796	1195	457	40.68
LNG-3	41,402	44,364,938	1071	1757	1173	447	40.62

Sample: Sample name; Total Number: The total number of Unigenes; Total Length: The read length of Unigenes; Mean Length: The average length of Unigenes; N50: The N50 length is used to determine the assembly continuity, the higher the better.N50 is a weighted median statistic that 50% of the total length is contained in transcripts that are equal to or larger than this value.; N70: Similar to N50; N90: Similar to N50; GC(%): the percentage of G and C bases in all Unigenes.

**Table A5:** Annotation results in different databases.

Database	Numbers of Unigene	Percent (%)
Total	112,543	100
Nr	66,783	59.34
Nt	52,701	46.83
Swiss-Prot	45,341	40.29
KEGG	51,648	45.89
KOG	55,832	49.61
Interpro	60,048	53.36
GO	36,323	32.27
Intersection	20,113	17.87
Overall	76,949	68.37

Intersection: The number of Unigenes which annotated by all the 7 functional databases; Overall: The number of Unigenes which annotated by any of the 7 functional databases. *Cut-off E-value ≤ 10^−5.^

**Table A6:** The top 10 enzymes with the largest differences in seven pathways for which gene shifts were significant.

	Functional Category/Subcategory	Down-Regulated	UpRegulated
pentose phosphate pathway	Pentose-phosphate shunt	thiamine biosynthesis protein ThiC	glucose-6-phosphate 1-dehydrogenase
		GTP-binding protein LepA	pyridoxine biosynthesis protein
		ATP-binding protein involved in chromosome partitioning	acetyltransferase NSI-like
		plastid-lipid-associated protein 12	transaldolase
		peptidylprolyl isomerase	oxidoreductase YkwC-like
		ATP-binding protein involved in chromosome partitioning	
		phospholipase D	
		ATP-dependent RNA helicase DDX56/DBP9	
		d-threo-aldose 1-dehydrogenase	
		tRNA (cytosine38-C5)-methyltransferase	
	Vitamin	farnesol kinase	vitamin E
		acyl-activating enzyme 14	vitamin B
		naphthoate synthase	
		MSBQ methyltransferase	
		tyrosine aminotransferase	
		glutamine amidotransferase	
		6-phosphogluconolactonase	
	Iron and sulfate metabolism	myo-inositol-1(or 4)-monophosphatase	ferritin heavy chain
		ATP synthase protein I	adenylylsulfate kinase
		transcription initiation factor TFIIF subunit alpha	
		polyadenylate-binding protein	
		cysteine desulfurase	
		prolyl-tRNA synthetase	
		bis(5′-adenosyl)-triphosphatase	
		(E)-4-hydroxy-3-methylbut-2-enyl-diphosphate synthase	
		BolA protein	
		selenocysteine lyase	
	small molecule metabolism	carbonic anhydrase	electron transfer flavoprotein beta subunit
		CUG-BP- and ETR3-like factor	3-oxoacyl-[acyl-carrier protein] reductase
		metal ion binding protein	betaine-aldehyde dehydrogenase
			COQ10 B, mitochondrial precursor
			4-hydroxybenzoate hexaprenyltransferase
			aldehyde dehydrogenase (NAD+)
			alcohol dehydrogenase
			omega-hydroxypalmitate O-feruloyl transferase
			AdoMet-dependent rRNA methyltransferase SPB1
			F-box protein 3
	Disease resistance proteins	Disease resistance protein RPM1	mlo protein
		MLO-like protein 4-like	disease resistance protein RPM1
		disease resistance protein At4g27190-like	disease resistance protein RPS5
		Disease resistance protein RFL1	
		disease resistance protein At4g27190-like	
		Disease resistance protein RPP13	
		disease resistance protein At1g61300-like	
		Disease resistance protein RPS2	
		MLO-like protein 13-like	
		disease resistance protein At4g27190-like	
	ATP-dependent proteases	ATP-dependent metalloprotease	ATP-dependent RNA helicase DHX8/PRP22
		ATP-dependent Clp protease	AFG3 family protein
		cell division protease FtsH	
		ATP-dependent Clp protease adaptor protein ClpS	
	Cysteine proteases	cathepsin L	cysteine protease
		cysteine protease inhibitor	
		cathepsin H	
	
		bark storage protein A-like	
		nudix hydrolase 8-like	
		cytosolic purine 5′-nucleotidase	
		nudix hydrolase 10	
		3-5 exonuclease	
		cytosolic purine 5′-nucleotidase	
Metabolism	Nucleoside metabolism	nudix hydrolase 20, chloroplastic-like	nucleoside-diphosphate kinase
		nitrilase homolog 1-like	ribonuclease Z
		adenine phosphoribosyltransferase	thyroid adenoma-associated protein homolog
		dihydropyrimidinase	uridine monophosphate synthetase
		callose synthase	GDPmannose 4,6-dehydratase
		centromeric protein E	ubiquitin carboxyl-terminal hydrolase 36/42
		dynein light chain LC6	ribose-phosphate pyrophosphokinase
		nucleoprotein TPR	elongation factor Tu
		actin 1	ribonucleoside-diphosphate reductase subunit M1
		microtubule-associated protein 70-5-like	adenosine kinase
	Structure	Rho GDP-dissociation inhibitor	tubulin beta
		kinesin family member C1	microfibrillar-associated protein 1
		dynein light chain LC8-type	tubulin gamma
		microtubule organization protein	Kinesin family member 11
		tubulin gamma	nuclear pore complex protein Nup62
		large subunit ribosomal protein L2	gelsolin
		small subunit ribosomal protein S16e	nuclear pore complex protein Nup62
		small subunit ribosomal protein S1	kinesin family member 15
		large subunit ribosomal protein L1	kinesin family member 22
		small subunit ribosomal protein S18	kinesin family member 18/19
		large subunit ribosomal protein L24	exportin-1
Protein synthesis	Ribosomal proteins	50S ribosomal protein 6	large subunit ribosomal protein LP1
		30S ribosomal protein S31	small subunit ribosomal protein S25e
		large subunit ribosomal protein L15	small subunit ribosomal protein S6e
		small subunit ribosomal protein S9	large subunit ribosomal protein L31e
		xial regulator YABBY 5	large subunit ribosomal protein L27Ae
		threonine-protein kinase/endoribonuclease	large subunit ribosomal protein LP0
		peptide chain release factor 1	large subunit ribosomal protein L37Ae
		auxin-responsive protein	large subunit ribosomal protein L13e
		peptide chain release factor 2	small subunit ribosomal protein S14e
		nucleic acid binding protein	small subunit ribosomal protein S21e
	Translation	peptidyl-tRNA hydrolase	ATP-dependent RNA helicase DDX6/DHH1
		translation initiation factor e	auxin-responsive protein
		endoribonuclease	translation initiation factor 3 subunit K
	Initiation	protein TIF31	translation initiation factor 5A
		eukaryotic translation initiation factor 3 subunit	translation initiation factor 4A
		farnesol dehydrogenase	translation initiation factor 2 subunit 3
		translation initiation factor 5A	translation initiation factor 4E
		transcription initiation factor TFIIE subunit beta	translation initiation factor eIF-2B subunit gamma
		translation initiation factor eIF-2B subunit beta	protein TIF31
		translation initiation factor IF-1	translation initiation factor 3 subunit I
		translation initiation factor 1	ATP-dependent RNA helicase
		KIAA0664 homolog	translation initiation factor 3 subunit D
		translation initiation factor IF-3	translation initiation factor 2 subunit 1
	Elongation	elongation factor G	elongation factor 2
		elongation factor P	elongation factor 1-alpha
			elongation factor 1-beta
			elongation factor Ts
			elongation factor G
			elongation factor P
	Others	small subunit ribosomal protein S6	small subunit ribosomal protein S24e
		NAD(P)H-quinone oxidoreductase subunit 2	large subunit ribosomal protein L10Ae
		thylakoid membrane organization	small subunit ribosomal protein S4e
		large subunit ribosomal protein L27	small subunit ribosomal protein S23e
		large subunit ribosomal protein L17	large subunit ribosomal protein L19e
		large subunit ribosomal protein L9	large subunit ribosomal protein L17e
		large subunit ribosomal protein L7/L12	large subunit ribosomal protein L8e
		large subunit ribosomal protein L18	large subunit ribosomal protein L39e
		small subunit ribosomal protein S13	small subunit ribosomal protein S2e
		Mitochondrial protein	small subunit ribosomal protein S15Ae
	Protein myristoylation	U6 small nuclear ribonucleoprotein PRP4	phosphoethanolamine *N*-methyltransferase
		glycylpeptide *N*-tetradecanoyltransferase	heat shock protein 90kDa beta
		protein *N*-terminal asparagine amidohydrolase	glycylpeptide *N*-tetradecanoyltransferase
		acylaminoacyl-peptidase	vacuolar protein 8
		kinesin family member C1	importin alpha
			E3 ubiquitin-protein ligase TRIP12
			stromal membrane-associated protein
			solute carrier family 44
			auxin response factor
Transport	Lipid and fatty acid transport	Cell wall-associated hydrolase	lipid binding protein
		phospholipid-translocating ATPase	non-specific lipid-transfer protein
		non-specific lipid-transfer protein 1-like	diazepam-binding inhibitor
		lipid transfer protein precursor	non-specific lipid-transfer protein 2-like
			phospholipid-translocating ATPase
			uncharacterized GPI-anchored protein At1g27950
			phospholipid-translocating ATPase
			uncharacterized protein LOC100305635 precursor
			tRNA wybutosine-synthesizing protein 3
			Niemann-Pick C1 protein
	vesicle-mediated and introcellular transport	syntaxin-41	synaptosomal-associated protein
		charged multivesicular body protein 3	FK506-binding nuclear protein
		Arf/Sar family	coatomer protein complex
		prolyl oligopeptidase	syntaxin-binding protein 1
		exocyst complex component 7	clathrin heavy chain
		ADP-ribosylation factor-like protein 5	FK506-binding nuclear protein
		phospholipase D	syntaxin 5
		vesicle-mediated transport	ESCRT-II complex subunit VPS36
		FK506-binding nuclear protein	oxidation resistance protein 1-like
		transforming growth factor-beta receptor-associated protein 1-like	Prenylated Rab acceptor protein
	Water transporters	solute carrier family 50	aquaporin PIP
		aquaporin NIP	aquaporin TIP
		aquaporin TIP	aquaporin-4
			aquaporin NIP
			3′-phosphoadenosine 5′-phosphosulfate synthase
			protein brassinosteroid insensitive 2
			aquaporin SIP
			solute carrier family 50
			exocyst complex component 7
	Gene silencing	arginine and glutamate-rich protein 1	interleukin-1 receptor-associated kinase 4
		RING finger and CHY zinc finger domain-containing protein 1	structural maintenance of chromosome 4
		THO complex subunit 3	kinesin family member 15
		symplekin	auxin response factor
		fused signal recognition particle receptor	cell division cycle 20-like protein 1
		protein MPE1	
		fused signal recognition particle receptor	chromatin assembly factor 1 subunit A
		UDP-*N*-acetylglucosamine pyrophosphorylase	copper chaperone
		RNA-binding protein 5/10	SNF-related matrix-associated actin-dependent regulator of chromatin subfamily A member 5
		kinesin family member C2/C3	LRR receptor-like serine/threonine-protein kinase FLS2
DNA replicaiton and cell cycle	DNA repair	deoxyribodipyrimidine photo-lyase	nucleosome assembly protein 1-like 1
		deoxyribonuclease V	dUTP pyrophosphatase
		riboflavin kinase	ATP-dependent RNA helicase DDX31/DBP7
		UDP-glucosyl transferase 73C	peptide-N4-(*N*-acetyl-beta-glucosaminyl)asparagine amidase
		BolA protein	DNA cross-link repair 1B protein
		heat shock protein HspQ	DNA repair protein RAD51
		peptide-N4-(*N*-acetyl-beta-glucosaminyl)asparagine amidase	deoxyribodipyrimidine photo-lyase
		DNA ligase 4	DNA-3-methyladenine glycosylase
		DNA mismatch repair protein MutS2	kinetochore protein Spc25
		DNA mismatch repair protein MutS2	DNA-3-methyladenine glycosylase II
	DNA replication	DNA polymerase eta subunit	GINS complex subunit 3
		leucine-rich repeat-containing protein 40-like	phospholipase D
		histone-binding protein RBBP4	high mobility group protein B1
		DNA primase small subunit	histone-binding protein RBBP4
		LRR receptor-like serine	DNA polymerase eta subunit
		replication factor C subunit 3/5	minichromosome maintenance protein 7
		DNA cross-link repair 1A protein	DNA polymerase alpha subunit A
		adenosylmethionine-8-amino-7-oxononanoate aminotransferase	DNA polymerase alpha subunit A
		E3 ubiquitin-protein ligase UBR7	thymidine kinase
			DNA primase large subunit
	DNA modification		DNA (cytosine-5-)-methyltransferase
			F-box protein, helicase, 18
	Other DNA metabolic processes	putative holliday junction resolvase	translation initiation factor 3 subunit M
			serine/arginine repetitive matrix protein 1
			ATP-dependent DNA helicase RecG
	Histones	enhancer of zeste	histone H2B
		ATP-binding cassette, subfamily F, member 2	histone H2A
		histone-lysine *N*-methyltransferase SETD1	nuclear transcription Y subunit beta
		histone deacetylase 6/10	histone-lysine *N*-methyltransferase SETD1
		histone H1/5	chromodomain-helicase-DNA-binding protein 4
		chromodomain-helicase-DNA-binding protein 4	enhancer of zeste
		[ribulose-bisphosphate carboxylase]-lysine *N*-methyltransferase	histone H3
		SAGA-associated factor 29	U4/U6.U5 tri-snRNP-associated protein 1
		enhancer of zeste	beta-1,3-galactosyltransferase
		histone acetyltransferase	nucleophosmin 1
	Chromasome structure maintenance	tRNA wybutosine-synthesizing protein 3	kinesin family member 18/19
		structural maintenance of chromosome 3	tRNA (cytosine38-C5)-methyltransferase
		DNA mismatch repair protein MLH3	structural maintenance of chromosome 4
		chromosome transmission fidelity protein 18	structural maintenance of chromosome 2
		high mobility group protein B1	DNA excision repair protein ERCC-6
		callose synthase	nijmegen breakage syndrome protein 1
		structural maintenance of chromosome 1	nucleosome assembly protein 1-like 1
		structural maintenance of chromosome 2	spastin
			structural maintenance of chromosome 3
			structural maintenance of chromosome 1
	Cell cycle regulation	ribonuclease P protein subunit POP4	protein neuralized
		microtubule-associated protein, RP/EB family	small subunit ribosomal protein S13e
		CCR4-NOT transcription complex subunit 1	FUS-interacting serine-arginine-rich protein 1
		cell cycle checkpoint protein	transitional endoplasmic reticulum ATPase
		FUS-interacting serine-arginine-rich protein 1	cyclin B
		pre-mRNA-splicing factor CWC22	bromodomain-containing factor 1
		cleavage and polyadenylation specificity factor subunit 2	E3 ubiquitin-protein ligase UHRF1
		CDK-activating kinase assembly factor MAT1	ubiquitin-conjugating enzyme E2 variant
		ER degradation enhancer, mannosidase alpha-like 1	ribonuclease P protein subunit POP4
		centromere/kinetochore protein ZW10	DNA polymerase alpha subunit B
Development	Cell growth	POZ domain-containing protein At2g30600	poly(A)-specific ribonuclease
		poly(A)-specific ribonuclease	adenylate isopentenyltransferase
		26S proteasome non-ATPase regulatory subunit 10	26S proteasome non-ATPase regulatory subunit 10
		preprotein translocase subunit YidC	ATP-dependent RNA helicase DDX46/PRP5
		activation cellfate specification	respiratory burst oxidase
		homeobox-leucine zipper protein	l-ascorbate oxidase
		root phototropism protein 3	peptidyl-prolyl isomerase G (cyclophilin G)
		sister chromatid cohesion protein PDS5	stress-induced-phosphoprotein 1
			respiratory burst oxidase
			alpha-l-fucosidase
	Tissue development	protein phosphatase 2	phospholipase D
		solute carrier family 31	nucleolin
		interleukin-1 receptor-associated kinase 4	solute carrier family 32 (vesicular inhibitory amino acid transporter)
		neutral invertase	regulator of Ty1 transposition protein 103
		multiple C2 and transmembrane domain-containing protein 2-like	small subunit ribosomal protein S10e
		E3 ubiquitin-protein ligase BRE1	carbamoyl-phosphate synthase large subunit
		histidyl-tRNA synthetase	calmodulin
		nucleolin	l-galactono-1,4-lactone dehydrogenase
		EID1-like F-box protein 3-like	26S proteasome regulatory subunit N12
		pleiotropic drug resistance protein 3-like	EIN3-binding F-box protein
	processes	cysteine synthase A	dynein light chain LC8-type
		protein-tyrosine phosphatase	protein-serine/threonine kinase
		NAC domain-containing protein	histone arginine demethylase JMJD6
		tropine dehydrogenase	methionyl aminopeptidase
		TCP4	interleukin-1 receptor-associated kinase 4
		beta-amyrin 24-hydroxylase	AP2-like factor, ANT lineage
		antiviral helicase SKI2	alpha-1,4-galacturonosyltransferase
		U3 small nucleolar RNA-associated protein 4	ubiquitin-conjugating enzyme E2 H
		phytochrome-interacting factor 3	two-component response regulator ARR-B family
			homeobox-leucine zipper protein
Stress response	Response to metal	tropine dehydrogenase	Ras-related protein Rab-1A
		ATP-binding cassette, subfamily B	jasmonate O-methyltransferase
		glutathione S-transferase	tyrosine 3-monooxygenase
		serine/threonine-protein kinase PBS1	aspartate aminotransferase
		ATP-binding cassette, subfamily B	alcohol dehydrogenase (NADP+)
		crt homolog 1	glutathione S-transferase
		circadian clock associated 1	d-threo-aldose 1-dehydrogenase
		pyridoxine 4-dehydrogenase	chaperonin GroEL
			CTP synthase
			copper-transporting atpase p-type
	Response to misfolded proteins	ubiquinol-cytochrome c reductase cytochrome c1 subunit	peptidyl-prolyl cis-trans isomerase A
		calmodulin	ubiquinol-cytochrome c reductase core subunit 1
		cell division cycle 20-like protein 1, cofactor of APC complex	cell division cycle 20-like protein 1
		DUF246 domain-containing protein At1g04910	F-type H+-transporting ATPase subunit delta
		ubiquitin-conjugating enzyme E2 D/E	aconitate hydratase 1
			mitotic spindle assembly checkpoint protein MAD2
			GTP-binding protein SAR1
			aspartate aminotransferase, cytoplasmic
			anaphase-promoting complex subunit 1
			cell cycle arrest protein BUB3
	Response to salts	aldose-6-phosphate reductase	beta-mannan synthase
		bromodomain-containing factor 1	V-type H+-transporting ATPase 21kDa proteolipid subunit
		bromodomain-containing protein 7/9	pectinesterase
		beta-mannan synthase	carboxymethylenebutenolidase
		sterile alpha motif and leucine zipper containing kinase AZK	hydroquinone glucosyltransferase
		outer membrane lipoprotein Blc	peroxidase
		AIG2-like	solute carrier family 15
		kynurenine formamidase-like	AP2-like factor, euAP2 lineage
		cyclin T	speckle-type POZ protein
		9-cis-epoxycarotenoid dioxygenase	extracellular signal-regulated kinase 1/2

Top 10 DEGs in every sub-pathway were listed according to the differences range from large to small. The enzymes in some pathways less than 10, because of an enzyme could regulate by multi-genes.

**Table A7:** Main DEGs in amino acid biosynthesis pathways (gall vs. LWG).

	Down-Regulated	Up-Regulated
Histidine	imidazoleglycerol-phosphate dehydratase	histidinol-phosphatase
		histidinol-phosphate aminotransferase
		imidazole glycerol-phosphate synthase subunit HisF
Tryptophan	anthranilate synthase / indole-3-glycerol phosphate synthase	tryptophan synthase alpha chain
	anthranilate synthase	anthranilate phosphoribosyltransferase
Tyrosine	cyclohexadieny/prephenate dehydrogenase	phenylalanine-4-hydroxylase
	Tyrosine aminotransferase	aromatic-amino-acid transaminase
		chorismate mutase
Phenylalaine		prephenate dehydratase
		shikimate kinase
		3-deoxy-7-phosphoheptulonate synthase
Serine		phosphoserine phosphatase
		phosphoserine aminotransferase
Cysteine	cysteine synthase	
	serine O-acetyltransferase	
Methionine		5-methyltetrahydrofolate--homocysteine methyltransferase
		cysteine-S-conjugate beta-lyase
		cystathionine beta-synthase
Alanine	alanine transaminase	
Glutamine	glutamine synthetase	
Arginine	argininosuccinate synthase	
Proline	glutamate 5-kinase	pyrroline-5-carboxylate reductase
		glutamate-5-semialdehyde dehydrogenase
Valine	acetolactate synthase I/II/III large subunit	l-serine dehydratase
		dihydroxy-acid dehydratase
		branched-chain amino acid aminotransferase
Leucine		2-isopropylmalate synthase
		3-isopropylmalate dehydrogenase
		branched-chain amino acid aminotransferase
Lysine		l-2-aminoadipate reductase
		kynurenine/2-aminoadipate aminotransferase
		diaminopimelate decarboxylase
		ll-diaminopimelate aminotransferase
		4-hydroxy-tetrahydrodipicolinate reductase
		4-hydroxy-tetrahydrodipicolinate synthase
		aspartate-semialdehyde dehydrogenase
		aspartate kinase

**Table A8:** Main DEGs in amino acid degradation pathways (gall vs. LWG).

	Down-Regulated	Up-Regulated
Tyrosine	tyrosine decarboxylase	
Phenylalaine	Phenylalaine dehydrogenase	
Serine		tryptophan synthase alpha chain
Cysteine	cysteine-S-conjugate beta-lyase	aspartate aminotransferase
	cystathionine gamma-lyase	
	S-adenosylmethionine decarboxylase	
Arginine		arginine decarboxylase
Proline	proline dehydrogenase	prolyl 4-hydroxylase
Threonine		l-serine/l-threonine ammonia-lyase
Glycine		glycine dehydrogenase

**Table A9:** Enzymes with the biggest difference in shikimate pathways (gall vs. LWG).

	Up-Regulated	Down-Regulated
Phenylpropanoid pathway	peroxidase	cinnamyl-alcohol dehydrogenase
	4-coumarate--CoA ligase	ferulate-5-hydroxylase
	5-O-(4-coumaroyl)-D-quinate 3′-monooxygenase	cinnamoyl-CoA reductase
	caffeic acid 3-O-methyltransferase	feruloyl-CoA 6-hydroxylase
	trans-cinnamate 4-monooxygenase	
	coniferyl-alcohol glucosyltransferase	
Flavonols	flavonoid 3′,5′-hydroxylase	flavonol 3-O-glucosyltransferase
	flavonoid 3′-monooxygenase	
	isoflavone 7-O-glucoside-6″-O-malonyltransferase	
	flavonol 3-O-methyltransferase	
Isoflavonols	2-hydroxyisoflavanone dehydratase	2-hydroxyisoflavanone synthase
	isoflavone-7-O-methyltransferase	isoflavone/4′-methoxyisoflavone 2′-hydroxylase
	2,7,4′-trihydroxyisoflavanone 4′-O-methyltransferase	vestitone reductase
	isoflavone 7-O-glucoside-6″-O-malonyltransferase	
	isoflavone 7-O-glucosyltransferase	
Anthocyanins	anthocyanidin 3-O-glucoside 2″′-O-xylosyltransferase	anthocyanidin 3-O-glucosyltransferase
	anthocyanidin 5,3-O-glucosyltransferase	

Enzymes were listed according to the differences range from large to small.

**Table A10:** Enzymes with the biggest difference in plant–pathogen interaction pathways (LNG vs. LWG).

Up-Regulated	Down-Regulated
LRR receptor-like serine/threonine-protein kinase FLS2	Di-glucose binding protein with Kinesin motor domain
disease resistance protein At4g27190-like	EIX receptor 1/2
WRKY transcription factor 2	calcium-dependent protein kinase 11
cyclic nucleotide-gated ion channel 1	disease resistance protein TAO1-like
heat shock protein 83	
disease resistance protein At4g27220	
disease resistance protein RPS2	

Enzymes were listed according to the differences range from large to small.

**Table A11:** The content of free amino acids and essential amino acids in different samples (Chao, 2020).

Samples	Free Amino Acids	Essential Amino Acids
Gall	3.098 + 0.001c	1.098 + 0.000c
LWG	2.928 + 0.001d	0.038 + 0.000d
LNG	3.230 + 0.001b	1.145 + 0.001b
aphids	4.796 + 0.002a	1.701 + 0.001a

## References

[1] Rohfritsch O., Shorthouse J.D. (1982). Insect Galls. Molecular Biology of Plant Tumors.

[2] Stone G.N., Karsten S. (2003). The adaptive significance of insect gall morphology. Trends Ecol. Evol..

[3] Tooker J.F., Helms A.M. (2014). Phytohormone Dynamics Associated with Gall Insects, and their Potential Role in the Evolution of the Gall-Inducing Habit. J. Chem. Ecol..

[4] Ferreira B.G., Álvarez R., Bragança G.P., Alvarenga D.R., Pérez-Hidalgo N., Isaias R.M.S. (2019). Feeding and Other Gall Facets: Patterns and Determinants in Gall Structure. Bot. Rev..

[5] Álvarez R., Encina A., Hidalgo N.P. (2009). Histological aspects of three *Pistacia terebinthus* galls induced by three different aphids: *Paracletus cimiciformis*, *Forda marginata* and *Forda formicaria*. Plant Sci..

[6] Stone G.N., Schönrogge K., Atkinson R.J., Bellido D., Pujade-Villar J. (2002). The Population Biology of Oak Gall Wasps (Hymenoptera: Cynipidae). Annu. Rev. Èntomol..

[7] Wool D. (2004). GALLIN GAPHIDS: Specialization, Biological Complexity, and Variation. Annu. Rev. Èntomol..

[8] Morris D.C., Schwarz M.P., Cooper S.J.B., Mound L.A. (2002). Phylogenetics of Australian Acacia thrips: The evolution of behaviour and ecology. Mol. Phylogenet. Evol..

[9] Blanche K.R. (2000). Diversity of insect-induced galls along a temperature- rainfall gradient in the tropical savannah region of the Northern Territory, Australia. Austral Ecol..

[10] Donald G.M.I., Ivey C.T., Shedd J.D. (2009). Support for the microenvironment hypothesis for adaptive value of gall induction in the California gall wasp, *Andricus quercuscalifornicus*. Èntomol. Exp. Appl..

[11] Alvarez R., Molist P., González-Sierra S., Martinez J.J.I., Nafrıa J.M.N. (2014). The histo structure of galls induced by aphids as a useful taxonomic character: The case of *Rectinasus* (Hemiptera, Aphididae, Eriosomatinae). Zootaxa.

[12] Costello J.F. (2001). Methylation matters. J. Med Genet..

[13] Martinez J.J.I. (2010). Anti-insect effects of the gall wall of *Baizongia pistaciae* [L.], a gall-inducing aphid on *Pistacia palaestina* Boiss. Arthropod Plant Interact..

[14] Shorthouse J.D., Wool D., Raman A. (2005). Gall-inducing insects—Nature’s most sophisticated herbivores. Basic Appl. Ecol..

[15] Nabity P.D., Haus M.J., Berenbaum M.R., DeLucia E.H. (2013). Leaf-galling phylloxera on grapes reprograms host metabolism and morphology. Proc. Natl. Acad. Sci. USA.

[16] Fay P.A., Hartnett D.C., Knapp A.K. (1993). Increased photosynthesis and water potentials in *Silphium integrifolium* galled by cynipid wasps. Oecologia.

[17] Moseley C.T., Cramer M.D., Hoffmann C.A.K.H. (2009). Why does Dasineura dielsi-induced galling of Acacia cyclops not impede vegetative growth?. J. Appl. Ecol..

[18] Andersen P.C., Mizell R.F. (1987). Physiological Effects of Galls Induced by *Phylloxera notabilis* (Homoptera: Phylloxeridae) on Pecan Foliage. Environ. Èntomol..

[19] Florentine S.K., Raman A., Dhileepan K. (2005). Effects of Gall Induction by Epiblema Strenuana on Gas Exchange, Nutrients, and Energetics in Parthenium Hysterophorus. BioControl.

[20] Wingler A., Roitsch T. (2008). Metabolic regulation of leaf senescence: Interactions of sugar signalling with biotic and abiotic stress responses. Plant Biol..

[21] Morkunas I., Mai V.C., Gabryś B. (2011). Phytohormonal signaling in plant responses to aphid feeding. Acta Physiol. Plant..

[22] Chen H., Liu J., Cui K., Lu Q., Wang C., Wu H., Yang Z., Ding W., Shao S., Wang H. (2018). Molecular mechanisms of tannin accumulation in Rhus galls and genes involved in plant-insect interactions. Sci. Rep..

[23] Yang Z. (2011). High Yield Cultivation Technology of Chinese Gallnut.

[24] Shao S.-X., Yang Z.-X., Chen X.-M. (2013). Gall development and clone dynamics of the galling aphid *Schlechtendalia chinensis* (Hemiptera: Pemphigidae). J. Econ. Èntomol..

[25] Chen X., Yang Z., Chen H., Qi Q., Liu J., Wang C., Shao S., Lu Q., Li Y., Wu H. (2020). A Complex Nutrient Exchange Between a Gall-Forming Aphid and Its Plant Host. Front. Plant Sci..

[26] Gonzalez P.S., O’Prey J., Cardaci S., Barthet V.J.A., Sakamaki J.-I., Beaumatin F., Roseweir A., Gay D.M., Mackay G., Malviya G. (2018). Mannose impairs tumour growth and enhances chemotherapy. Nature.

[27] Cai W., Pang X., Hua B., Liang G., Song D. (2001). Basic Entomology.

[28] French D. (1973). Chemical and Physical Properties of Starch. J. Anim. Sci..

[29] Nowak H., Komor E. (2010). How aphids decide what is good for them: Experiments to test aphid feeding behaviour on *Tanacetum vulgare* (L.) using different nitrogen regimes. Oecologia.

[30] Wang C., Chen X.-M., Yang Z.-X., Chen H., Shao S.-X., Wu H.-X. (2018). Studies on free amino acids of aphid *Schlechtendalia chinensis* and the host plant *Rhus chinensis*. For. Res..

[31] Wu H.-X., Chen X., Chen H., Lu Q., Yang Z., Ren W., Liu J., Shao S., Wang C., King-Jones K. (2018). Variation and diversification of the microbiome of *Schlechtendalia chinensis* on two alternate host plants. PLoS ONE.

[32] Mashiguchi K., Hisano H., Takeda-Kamiya N., Takebayashi Y., Ariizumi T., Gao Y., Ezura H., Sato K., Zhao Y., Hayashi K.-I. (2019). *Agrobacterium tumefaciens* Enhances Biosynthesis of Two Distinct Auxins in the Formation of Crown Galls. Plant Cell Physiol..

[33] Zhang H., Lang Z., Zhu J.-K. (2018). Dynamics and function of DNA methylation in plants. Nat. Rev. Mol. Cell Biol..

[34] Rawat N., Neeraja C.N., Nair S., Bentur J.S. (2012). Differential gene expression in gall midge susceptible rice genotypes revealed by suppressive subtraction hybridization (SSH) cDNA libraries and microarray analysis. Rice.

[35] Zhu L., Liu X., Liu X., Jeannotte R., Reese J.C., Harris M., Stuart J.J., Chen M.-S. (2007). Hessian Fly (Mayetiola destructor) Attack Causes a Dramatic Shift in Carbon and Nitrogen Metabolism in Wheat. Mol. Plant Microbe Interact..

[36] Gonzáles W.L., Fernández R. (2000). Do host plant traits and galling insects affect the abundance of *Issus* sp. on *Colliguaja odorifera* Mol. along an altitudinal gradient?. Rev. Chil. Entomol..

[37] Guo R., Wang Y.P., Hong W.U. (2012). The diversity of insects galls and relationships between insects galls and their host plants and environment. J. Environ. Entomol..

[38] Lu Q., Chen H., Wang C., Yang Z.-X., Lü P., Chen M.-S., Chen X.-M. (2019). Macro- and Microscopic Analyses of Anatomical Structures of Chinese Gallnuts and Their Functional Adaptation. Sci. Rep..

[39] Larson K.C., Whitham T.G. (1991). Manipulation of food resources by a gall-forming aphid: The physiology of sink-source interactions. Oecologia.

[40] Grabherr M.G., Haas B.J., Yassour M., Levin J.Z., Thompson D.A., Amit I., Adiconis X., Fan L., Raychowdhury R., Zeng Q. (2011). Full-length transcriptome assembly from RNA-Seq data without a reference genome. Nat. Biotechnol..

